# JMJD-1.2 controls multiple histone post-translational modifications in germ cells and protects the genome from replication stress

**DOI:** 10.1038/s41598-018-21914-9

**Published:** 2018-02-28

**Authors:** Toshia R. Myers, Pier Giorgio Amendola, Yvonne C. Lussi, Anna Elisabetta Salcini

**Affiliations:** 1 Biotech Research & Innovation Centre (BRIC), University of Copenhagen, Ole Maaløes Vej 5, DK-2200 Copenhagen N, Denmark; 2 Centre for Epigenetics, University of Copenhagen, Ole Maaløes Vej 5, DK-2200 Copenhagen N, Denmark

## Abstract

Post-translational modifications of histones, constitutive components of chromatin, regulate chromatin compaction and control all DNA-based cellular processes. *C*. *elegans* JMJD-1.2, a member of the KDM7 family, is a demethylase active towards several lysine residues on Histone 3 (H3), but its contribution in regulating histone methylation in germ cells has not been fully investigated. Here, we show that *jmjd-1*.*2* is expressed abundantly in the germline where it controls the level of histone 3 lysine 9, lysine 23 and lysine 27 di-methylation (H3K9/K23/K27me2) both in mitotic and meiotic cells. Loss of *jmjd-1*.*2* is not associated with major defects in the germ cells in animals grown under normal conditions or after DNA damage induced by UV or ionizing irradiation. However, *jmjd-1*.*2* mutants are more sensitive to replication stress and the progeny of mutant animals exposed to hydroxyurea show increased embryonic lethality and mutational rate, compared to wild-type. Thus, our results suggest a role for *jmjd-1*.*2* in the maintenance of genome integrity after replication stress and emphasize the relevance of the regulation of histone methylation in genomic stability.

## Introduction

The eukaryotic genome is organized in the nucleus as chromatin, a dynamic structure composed mainly of DNA and histone proteins. Post-translational modifications of histone amino-terminal tails influence chromatin organization and control transcriptional activity and other DNA-based cellular processes, including DNA replication and responses to DNA damage^1,2^. Lysine methylation is one of many histone modifications that has been widely studied^3^. Mutations in genes encoding for histone lysine methyltransferases (KMTs) and histone lysine demethylases (KDMs), enzymes that deposit and remove methyl groups, respectively, are associated with several diseases including cancer^4–8^. While the role of histone lysine methylation in regulating transcription has been described in some detail, less is known about lysine methylation during DNA replication and replication stress, in particular at the organismal level. During replication, DNA is subject to different sources of stress that can result in DNA damage and genomic instability^9,10^. As methylated histones are enriched at replication sites, KMTs and KDMs are emerging as regulators of replication^11^, with a potential role in the maintenance of genome stability.

Genome stability is particularly important in germ cells to ensure fertility and prevent defects that can be stably transferred to progeny, thus negatively influencing the fitness of subsequent generations. The *C*. *elegans* germline provides a unique context to study the regulation of histone post-translational modifications as well as their function in germ cells and transgenerational impact. We and others previously identified *C*. *elegans* JMJD-1.2, a component of the mammalian KDM7 demethylase family and homologue to the mammalian PHF8, as a H3K9/K23/K27me2 demethylase^8,12–14^. In *C*. *elegans*, JMJD-1.2 is required in somatic cells for correct neuronal function^12^, and its loss is associated with defects in axon migration^15^. However, the contribution of JMJD-1.2 in the control of histone methylation in germ cells has not been fully elucidated. Here, we characterize the role of *jmjd-1*.*2* in germ cells. Our results suggest that JMJD-1.2 acts as a demethylase for H3K9/23/27me2 in germ cells and contributes to the maintenance of genome integrity after replication stress.

## Results

### Localization of JMJD-1.2 in germ cells

**Figure 1:**
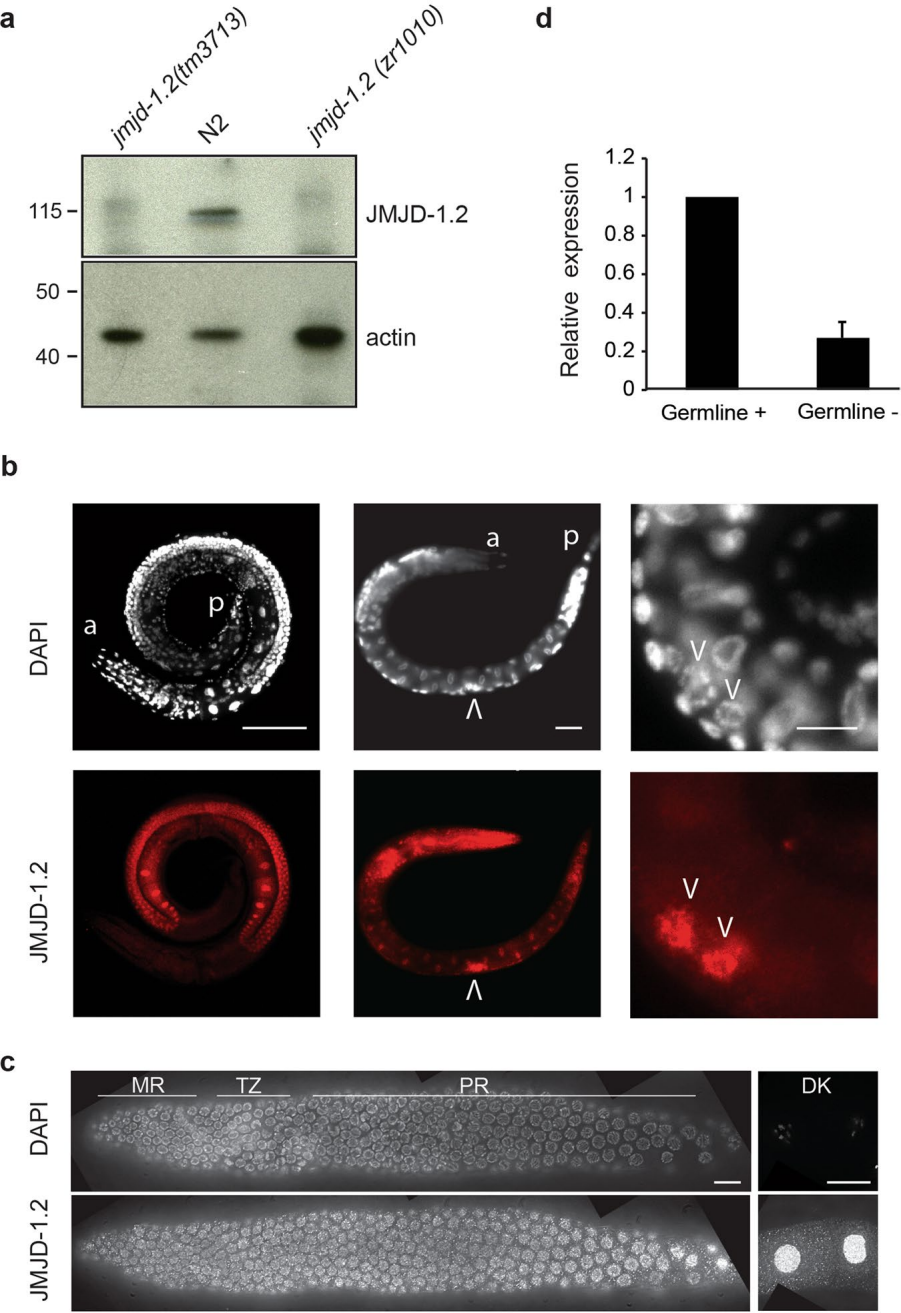
JMJD-1.2 is expressed in the germline. (**a**) Representative western blot analysis of lysates extracted from the indicated genotypes using JMJD-1.2 antibody. Actin is used as loading control. (**b**) Representative images of wild-type (N2) animals (adult, left panel; L1 stage, middle and right panels) stained with JMJD-1.2 specific antibody (lower panels) and DAPI staining (upper panels). a, anterior part of the animals, p, posterior part of the animal. Arrowheads indicate the precursor germ cells at L1 stage. Scale bars, 100 μm (left panel) and 10 μm (middle and right panels). (**c**) Germline excised from N2 young adult hermaphrodite, reconstructed using ImageJ. The mitotic region is on the left and oocytes are in separate panels on the far right. The top panel shows DAPI staining and the bottom panel anti-JMJD-1.2 staining. 100× magnification; scale bar, 10 μm. MR, mitotic region; TZ, transition zone; PR, pachytene region, DK, oocytes in diakinesis. (**d**) Relative expression of *jmjd-1*.*2* measured by quantitative PCR using *glp-4*(*bn2*), grown at 20 °C (gemline+) and at the restrictive temperature of 25 °C (germline −), in which the gonads are absent. cdc-42 is used as internal control. Bar indicates SD from three independent experiments.

*jmjd-1*.*2* encodes a protein containing a JmjC domain that demethylates H3K9me2, H3K27me2, and H3K23me2 and a PHD finger domain that interacts with H3K4me3^12–14^. To investigate whether *jmjd-1*.*2* functions in germ cells, we utilized two deletion alleles: *tm3713* carrying a deletion of the PHD domain and *zr1010*, a CRISPR-engineered knockout that removes the entire coding sequence^15^. Western blot analysis of wild-type (N2) lysates with a polyclonal antibody against the N-terminus of JMJD-1.2 showed a band of approximately 120 KDa that was absent in mutant lysates (Figs 1a and S1), demonstrating the specificity of the antibody for JMJD-1.2. Immunostaining of whole animals with the specific antibody showed that a sizable fraction of JMJD-1.2 is located in adult germ cells as well as in the precursor germ cells of young animals (Figs 1b and S2). In excised gonads, JMJD-1.2 localized to the nucleoplasm throughout the entire germline, in particular in oocytes (Fig. 1c). In agreement, quantitative PCR analysis in germline-depleted animals suggested that *jmjd-1*.*2* is expressed in germ cells (Fig. 1d). Overall, these results indicate that JMJD-1.2 is strongly expressed in the germline at different stages of germ cell development.

### JMJD-1.2 regulates multiple post-translational modifications in germ cells but its loss does not impair fundamental germline functions

**Figure 2:**
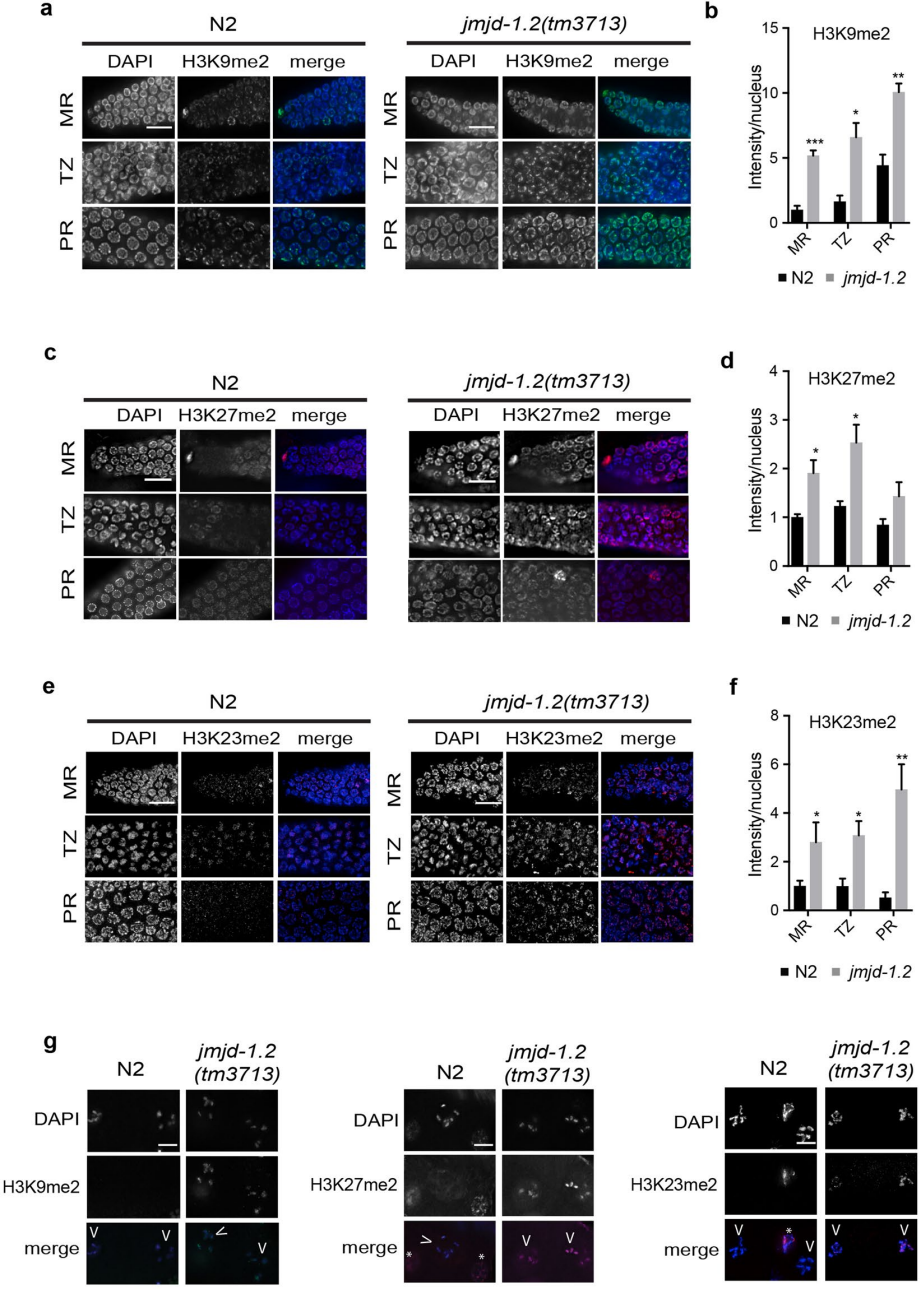
JMJD-1.2 is required for H3K9/K23/27me2 modulation. (**a**) Representative images of indicated germline regions of N2 (left) and *jmjd-1*.*2*(*tm3713*) (right) stained with DAPI (blue) and anti-H3K9me2 (green). (**b**) Quantification of the average H3K9me2 intensity per nuclei. (**c**) Representative images of indicated germline regions of N2 and *jmjd-1*.*2*(*tm3713*) stained with DAPI (blue) and anti-H3K27me2 (red). (**d**) Quantification of the average H3K27me2 intensity per nuclei. (**e**) Representative images of indicated germline regions of N2 and *jmjd-1*.*2*(*tm3713*) stained with DAPI (blue) and anti-H3K23me2 (red). (**f**) Quantification of the average H3K23me2 intensity per nuclei. In b, d and f, the fluorescence intensity is expressed in arbitrary units, relative to the intensity of the N2 strain measured at the mitotic region and set at 1. Data are presented as mean +/− SEM. *p ≤ 0.05, **p ≤ 0.01, ***p ≤ 0.001, with two-tailed unpaired *t*-test. Regions derived from 3 to 8 gonads were used for the quantification. (**g**) Representative images of N2 and *jmjd-1*.*2*(*tm3713*) oocytes in diakinesis, stained with the indicated antibodies. Arrowheads indicate the oocytes and asterisks somatic cells. MR, mitotic region; TZ, transition zone; PR, pachytene region. Scale bar in (**a**,**c** and **e**) 10 μm, scale bar in (**g**) 5 μm.

To determine if JMJD-1.2 regulates H3K9/K23/K27me2 in germ cells, immunofluorescence (IF) was used to compare the levels of these marks in wild-type and *jmjd-1*.*2*(*tm3713*) germlines. As previously reported in wild-type germlines^16^, H3K9me2 and H3K27me2 were detected in all nuclei of the mitotic region, transition zone, and pachytene region (Fig. 2a–d) but disappeared in oocyte nuclei, when chromosomes are highly condensed in diakinesis (Fig. 2g). Similarly, H3K23me2 was detected as a faint and punctate staining in all germ cell nuclei and absent in oocyte nuclei (Fig. 2e–g and^14^). In *jmjd-1*.*2*(*tm3713*), the intensity of H3K9me2, H3K23me2, and H3K27me2 was increased in the nuclei of all germline regions, in comparison to wild-type, and all of the marks were detected in oocyte nuclei (Fig. 2a–g). The levels and localization of H4K20me1, a post-translational modification regulated by the *jmjd-1*.*2* mammalian homologue, PHF8^17^, were similar in both the wild-type and mutant germlines – at least at the level of detection of IF (Fig. S3). These results indicate that JMJD-1.2 acts in the *C*. *elegans* germline primarily as an H3K9me2, H3K23me2, and H3K27me2 demethylase.

**Figure 3:**
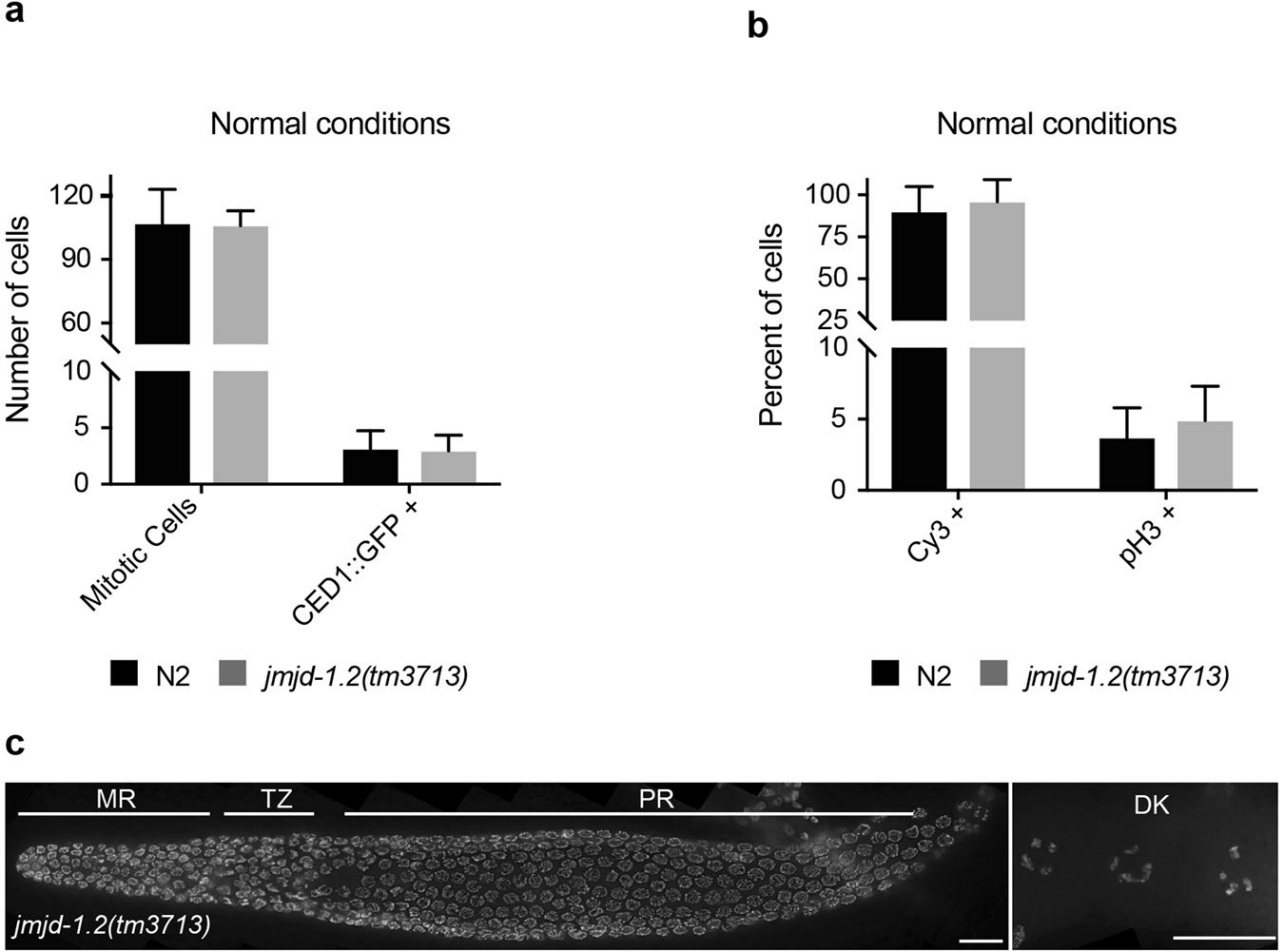
*jmjd*-*1*.*2* is not required for mitotic cell division and apoptosis. (**a**) Average number of mitotic cells and CED-1::GFP positive cells in N2 and *jmjd-1*.*2*(*tm3713*), grown in normal conditions. Data are from at least 15 gonads. (**b**) Percentage of Cy3-dUTP and pH3 positive mitotic nuclei, in N2 and *jmjd-1*.*2*(*tm3713*), grown in normal conditions. Data are from at least 6 gonads and 15 gonads, respectively. In a and b, bars indicate SD. No significant differences were observed with two-tailed paired *t*-test (p > 0.1). (**c**) Representative image of *jmjd-1*.*2*(*tm3713*) extracted gonad stained with DAPI. MR, mitotic region; TZ, transition zone; PR, pachytene region; DK, oocytes in diakinesis. Scale bars, 10 μm.

We next assessed the function of JMJD-1.2 in germ cells. Despite increased H3K9/K23/K27me2 levels, *jmjd-1*.*2* mutant animals were phenotypically wild-type for fundamental germline functions. Both *jmjd-1*.*2* mutant strains were fertile, with only a minor reduction of the brood size [mean +/− SD, n ≥ 7, N2: 257.9 +/− 44, *jmjd-1*.*2*(*tm3713*): 198.7 +/− 15, *jmjd-1*.*2*(*zr1010*): 215.7 +/− 26]. Embryonic lethality [N2: 0.2%, *jmjd-1*.*2*(*tm3713*): 0.25%, *jmjd-1*.*2*(*zr1010*): 0.3%, n > 1000] and male production [N2: 0.07%, *jmjd-1*.*2*(*tm3713*): 0.15%, *jmjd-1*.*2*(*zr1010*): 0.07%, n > 1000] were also unaffected in *jmjd-1*.*2* mutants. Microscopic analysis of germlines from *jmjd-1*.*2*(*tm3713*) showed no obvious physiological defects under normal growth conditions. *jmjd-1*.*2*(*tm3713*) germlines had a normal number of mitotic cells (Fig. 3a) and a normal rate of mitotic division, as measured by Cy3-dUTP incorporation and phosphorylated H3 (pH3) staining^18,19^ (Fig. 3b). Additionally, the level of physiological apoptosis, analyzed using the CED-1::GFP construct, was similar to the level observed in wild-type germlines (Fig. 3a) and all stages of meiosis were clearly identifiable in *jmjd-1*.*2*(*tm3713*) (Fig. 3c). Overall, these results indicate that, under normal growth conditions, deregulation of the histone post-translational modifications observed in the germline of *jmjd-1*.*2* mutants does not cause significant germline abnormalities.

### *jmjd-1*.*2* mutant animals are hypersensitive to replication stress

**Figure 4:**
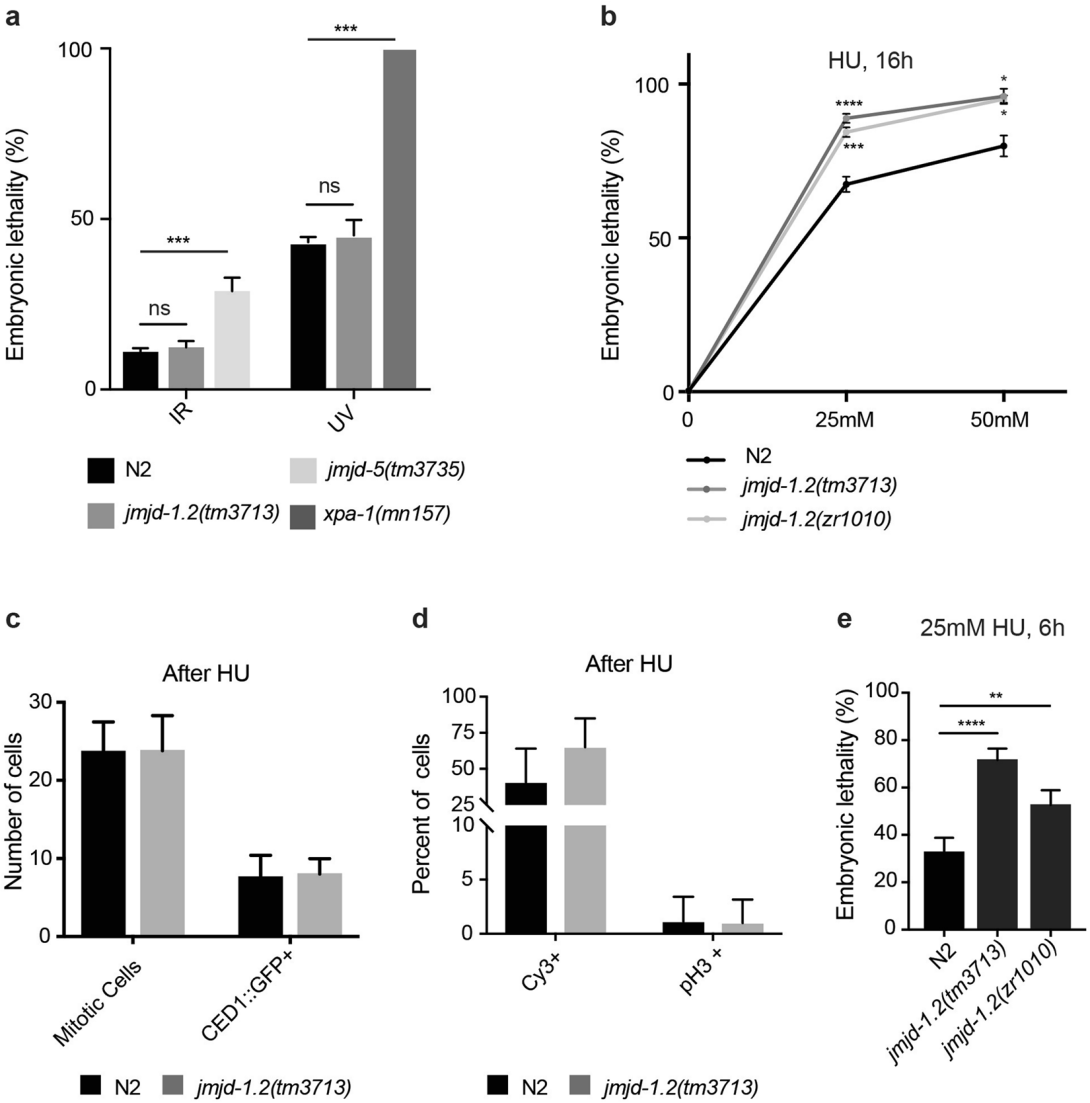
*jmjd*-*1*.*2* mutants are hypersensitive to HU. (**a**) Quantification of the percentage of embryonic lethality in the indicated genotypes in response to radiation (ionizing radiation, IR, 80 Gy; ultraviolet, UV, 300 j/m^2^). *jmjd-5*(*tm3735*) and *xpa-1*(*mn157*) mutant alleles are used as positive controls in IR and UV tests, respectively. (**b**) Quantification of the percentage of embryonic lethality in the indicated genotypes in response to different doses of hydroxyurea (HU) for 16 h. In **a** and **b**, the graphics are the average of at least 3 independent experiments and data are presented as mean +/− SEM. ns, no significant differences, *p ≤ 0.05, ***p ≤ 0.001, ****p ≤ 0.0001, comparing the mutant alleles with N2 with paired *t-*test. (**c**) Average number of mitotic cells and CED-1::GFP positive cells in N2 and *jmjd-1*.*2*(*tm3713*), grown in 25 mM HU for 16 hours. Data are from at least 15 gonads. (**d**) Percentage of Cy3-dUTP and pH3 positive mitotic nuclei, in N2 and *jmjd-1*.*2*(*tm3713*), grown in 25 mM HU for 16 hours. Data are from at least 6 gonads and 15 gonads, respectively. In c and d, bars indicate SD. No significant differences were observed with two-tailed paired *t*-test (p > 0.1). (**e**) Quantification of the percentage of embryonic lethality in the indicated genotypes in response to exposure to 25 mM HU for 6 hours. The graphic is the average of at least 3 independent experiments and data are presented as mean +/− SEM. **p ≤ 0.01, ****p ≤ 0.0001, with paired *t-*test.

*C*. *elegans* is an established model to examine DNA stress response *in vivo*^20^, and we utilized this system to determine if the loss of *jmjd-1*.*2*, and the resulting abnormal germline levels of H3K9/K23/K27me2, affects responses to DNA damaging agents. The amount of embryonic lethality (number of dead eggs/total number of progeny) in the progeny of hermaphrodites exposed to exogenous stressors was used as an indicator of impaired response^20^. We tested ionizing radiation (IR), which can activate the homologous recombination (HR) repair pathway in germ cells^21^ and ultraviolet-C radiation (UV-C), which primarily activates the nucleotide excision repair (NER) pathway^22^. Adult *jmjd-1*.*2*(*tm3713*) animals had a wild-type response to both IR (80 Gy) and UV-C (300 J/m^2^) exposure (Fig. 4a). We also tested the response of *jmjd-1*.*2* mutant animals to hydroxyurea (HU). HU inhibits ribonucleotide reductase and, by decreasing the production of deoxyribonucleotides, perturbs DNA replication^23–25^. In adult animals, this occurs exclusively in the mitotic cells located at the distal region of the germline. *jmjd-1*.*2*(*tm3713*) and *jmjd-1*.*2*(*zr1010*) mutants showed significantly increased embryonic lethality after HU treatments (25 and 50 mM) for 16 hours (Fig. 4b), suggesting that JMJD-1.2 is involved in replication stress protection. We further examined the role that JMJD-1.2 plays in replication stress response in mitotic germ cells. Similar to wild-type, *jmjd-1*.*2*(*tm3713*) mitotic germ cells exposed to HU were reduced in number and appeared enlarged in size (Fig. 4c, compare to Figs 3a and S4a), indicating a normal mitotic arrest. The number of cycling mitotic cells was also examined measuring the level of incorporated Cy3-dUTP and phosphorylated H3 (pH3), markers for DNA synthesis and mitotic cell division, respectively^18,19^. We found a similar reduction in Cy3-dUTP incorporation and pH3 expression in both mutant and wild-type mitotic cells (Fig. 4d, compare to Fig. 3b). Notably, Cy3-dUTP-positive cells progressed only to the pachytene stage when wild-type and mutant animals were treated with HU for 20 hours (Fig. S4b), indicating that the embryonic lethality observed after 16 hours of HU treatment is not due to the effect of HU on mitotic cells.

Prolonged stalling at replication forks results in DNA double-strand breaks and activation of apoptosis^26,27^ and could potentially affect embryonic lethality rates. Rates of apoptosis were measured using the CED-1:GFP marker^28,29^ after 16 hours HU treatment and no discernable difference between wild-type and mutant germlines was observed (Fig. 4c). Accordingly, similar to wild-type, the transcription level of *egl-1* gene, a CEP-1/P53 target, is elevated after HU treatment in *jmjd-1*.*2* mutant animals, indicating a normal activation of apoptosis (Fig. S5a). Likewise, RPA-1 and RAD-51 are properly recruited at DNA damage sites after HU treatment (Fig. S5b and c). Thus, both mitotic (cell cycle arrest) and meiotic (DNA damage repair and apoptosis) responses to HU treatment appears to occur normally in *jmjd-1*.*2* mutant animals, indicating that the observed embryonic lethality is not most likely related to aberrant germline reactions to HU. As HU treatment of adult animals has been also used to deliver HU to embryos, where it interferes with early embryonic cell divisions^30,31^, we hypothesized that *jmjd-1*.*2* could be required in the embryo for proper cell divisions. We therefore examined the effect of a shorter HU exposure (6 hours) on embryonic lethality and observed a significant increase in embryonic lethality in both mutant strains in comparison to wild-type animals (Fig. 4e), strongly suggesting that JMJD-1.2 acts to protect embryos from replication stress.

### JMJD-1.2 is required for genome stability after DNA replication stress

**Figure 5:**
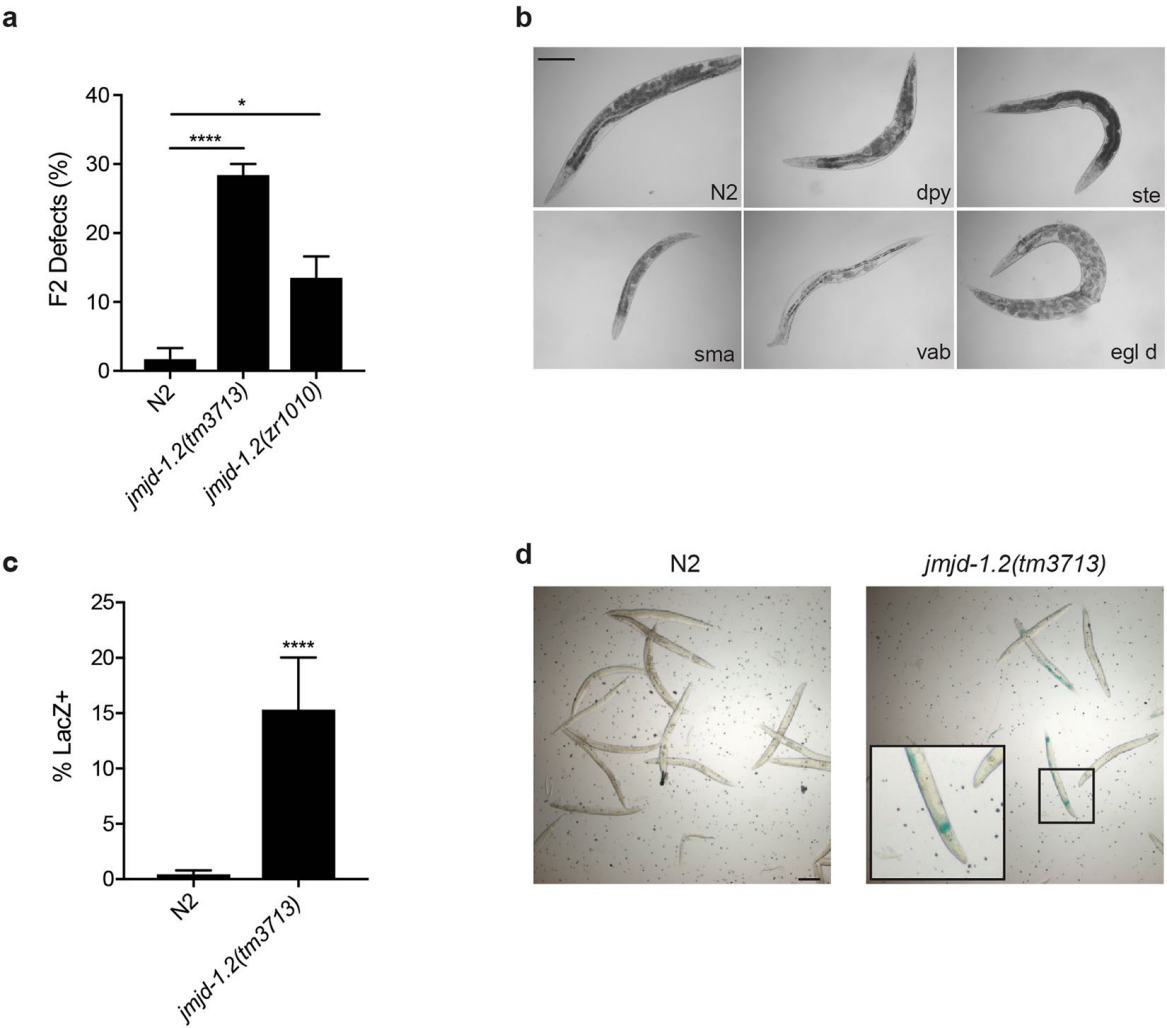
*jmjd-1*.*2* mutants have increased mutational rate after HU. (**a**) Quantification of the percentage of abnormalities identified in F2 progeny derived from animals of indicated genotypes exposed to HU (25 mM, 16 h). The percentage of F2 abnormalities was determined based on the number of plates that had at least one F2 abnormal animal. (**b**) Representative images of *jmjd-1*.*2* F2 progeny derived from animals exposed to HU (25 mM, 16 h). Dpy, dumpy; ste, sterile; sma, small; vab, organism morphology variant; egl d, egg laying defective. A wild-type animal (N2) is shown, for comparison. (**c**) Quantification of the percentage of *lacZ-* positive animals in F1 generation after parental HU treatment (25 mM, 16 h). N2 and *jmjd-1*.*2*(*tm3713*) carrying the pkIs1604 transgene are used. (**d**) Representative images of the F1 generation after parental HU treatment, (25 mM, 16 h) stained by X-gal. N2 and *jmjd-1*.*2*(*tm3713*), carrying the pkIs1604 transgene are shown. A magnification of a *lacZ-*positive animal is shown. In a and c, the graphics show the average of two independent experiments and data are presented as mean +/− SD. *p ≤ 0.05, ****p ≤ 0.0001, with chi square test. Scale bars, 100 μm.

To further investigate the effects of loss of *jmjd-1*.*2* in embryos generated after parental HU exposure, we cloned the surviving progeny (F1) of wild-type and *jmjd-1*.*2* mutant animals exposed to HU and followed them for one generation. We found that the F2 progeny derived from *jmjd-1*.*2* mutants exposed to HU showed a significant increase of visible phenotypes (e.g., Dpy, Rol, Unc, Pvl, Ste, Egl, Vab, Him) (Fig. 5a and b) in comparison to wild-type. These results suggest that *jmjd-1*.*2* mutants have increased mutation frequency after replication stress, in comparison to wild-type animals. To confirm this hypothesis, we measured the rate of F1 mutations generated after parental exposure to HU using a *lacZ* reporter assay that detects small DNA insertions and deletions. This reporter assay was previously used to identify genes implicated in genome protection in somatic and germ cells^32^ and has the potential to reveal heterozygous mutations. The *lacZ* reporter construct is out of frame and genetic alterations that bring the construct back in frame can be visualized in the animal as blue patches after X-gal staining. We did not observe X-gal staining in either wild-type or *jmjd-1*.*2*(*tm3713*) animals under normal growth conditions, further indicating that *jmjd-1*.*2* is not required for DNA stability in the absence of stress. However, after parental exposure to HU, blue patches were observed in F1 progeny of *jmjd-1*.*2*(*tm3713*), but not in wild-type F1 progeny (Fig. 5c and d). These data indicate that the F1 progeny of HU-exposed *jmjd-1*.*2* mutants have increased mutation rates.

Overall, our results suggest a role of JMJD-1.2 in the maintenance of genome integrity after replication stress.

## Discussion

In this study, we analyzed the presence and the enzymatic activity of JMJD-1.2 in germ cells. Our results indicate that JMJD-1.2 is abundantly expressed in germ cells where it acts as a demethylase for H3K9me2, H3K23me2, and H3K27me2. Methylated H3K9 and H3K27 are post-translational modifications characteristic of silenced chromatin and methylated H3K9 acts as a docking site for heterochromatic proteins (HPs), promoting the formation of heterochromatin^33,34^. H3K23me2, particularly abundant in *C*. *elegans*, has been proposed as a signature mark for heterochromatin as it co-occurs with methylated H3K9 and K27 and can be bound by HP-like proteins^14^. Compared to wild-type animals, *jmjd-1*.*2* mutants have higher levels of di-methylated H3K9/23/27 in all regions of the germline, including in the oocytes, where these marks are normally not detected. Despite this deregulation, there were no observable germline-associated defects, apart from a minor effect on the fertility, suggesting that germ cells can tolerate variations of histone modification levels, at least when grown under laboratory conditions.

However, the progeny of *jmjd-1*.*2* mutants exposed to replication stress have increased embryonic lethality, apparently not associated to defects in DNA damage response mechanisms in the germline. This result suggests that JMJD-1.2 could have a protective function against replication stress during early embryonic development. This possibility is supported by the increased embryonic lethality observed in *jmjd-1*.*2* animals after short HU exposure. This hypersensitivity to HU could be linked to an embryonic function of JMJD-1.2 in safeguarding DNA replication during early embryonic cell divisions or it could also be that the aberrant level of H3K9/23/27me2 observed in oocytes are passed on to embryos, impairing the embryonic replication stress response mechanisms^35,36^. Thus, further research on the effect of JMJD-1.2 in embryos is required to clarify the focus of action and the function of JMJD-1.2. Interestingly, *jmjd-1*.*2* mutant animals also exhibit an aberrant response to interstrand DNA crosslinks that lead to stalled DNA replication^37^, highlighting the role of JMJD-1.2 in mechanisms protecting DNA from different sources of replication stress.

Another *C*. *elegans* H3K9/K36me3 demethylase, JMJD-2, was previously implicated in DNA replication^38^. However, *jmjd-2* loss is associated with several defects in germ cells under normal conditions, such as reduced mitotic cells and increased level of RAD-51 foci, not observed in *jmjd-1*.*2* mutants. This evidence suggests a more specific action of *jmjd-1*.*2* when DNA replication is challenged, at least in nematode. Indeed, it is important to note that PHF8, the mammalian homologue of *jmjd-1*.*2*, has been previously implicated in cell cycle regulation. PHF8 is highly expressed in G2/M phases and its reduction by siRNAs decreases cell proliferation and results in delayed G1/S transition, prolonged G2 phase, and defective mitosis^17,39^. Yet, this cell cycle-associated function of PHF8 has thus far only been linked to its catalytic activity on H4K20me1, that we did not observe in the nematode. While it is remarkable that a single enzyme has the ability to remove marks associated with heterochromatin, the capacity of JMJD-1.2 to control the methylation of multiple histone residues makes it difficult to distinguish the affect that each residue has, either alone or in combination, on the phenotypes associated with loss of *jmjd-1*.*2* and to test possible mechanism(s) of action. Nevertheless, the evidence that loss of *jmjd-1*.*2* results in increased mutation rate after replication stress in association with deregulated levels of heterochromatic marks supports the notion that heterochromatin regulation is necessary for maintenance of genomic stability, as shown in other organisms. For example, loss of H3K9 methyltransferases leads to aneuploidy and meiotic defects in mice^40^ and in chromosomal translocations and loss of heterozygosity in *Drosophila*^41^. *Drosophila* mutants for the H3K9 methyl-binding protein HP1 show chromosome segregation defects and telomere fusion^42^. More recently, a role for heterochromatin in silencing repetitive elements has been proposed in *C*. *elegans*^43,44^ and *Drosophila*^45^, indicating that heterochromatin formation and maintenance can affect genome stability at multiple levels.

Although further experiments are required to explore the impact of a genome-wide loss of JMJD-1.2 on the post-translational modification landscape and the transcription of coding and non-coding genes, our study indicates that JMJD-1.2 ensures genome integrity after replication stress. The novel function of JMJD-1.2 here reported provides additional insight into how the human homologue PHF8 might contribute to different types of cancer^46–48^.

## Experimental Procedures

### *C*. *elegans* culture and Strains

Animals were grown at 20 °C on NGM plates seeded with OP50 *E*. *coli* bacteria under standard laboratory conditions^49^ unless otherwise indicated. In this study we used *jmjd-1*.*2*(*tm3713*) and *jmjd-1*.*2*(*zr1010*) alleles, previously described^15^. We noticed that the *jmjd-1*.*2*(*tm3713*) allele shows, in some cases, more penetrant phenotypes compared to the *jmjd-1*.*2*(*zr1010*) allele. As the (*tm3713*) allele carries an in-frame deletion, it is possible that this allele is not a complete null. Other strains used were: *jmjd-5*(*tm3735*), XP482 [*xpa-1*(*mn157*)], SS104 [*glp-4*(*bn2*)], MD701 {bcIs39 [lim-7p::ced-1::GFP + lin-15(+)]}, NL3400 {(pkIs1604 [hsp-16.2::ATG(A)17GFP::LacZ+ rol-6(su1006)]}, WS4581{opIs263[RPA-1p::RPA-1::YFP + unc-119(+)]}. N2 Bristol is the wild-type strain.

### Western blot

Whole-worm lysates for SDS-PAGE were prepared by boiling mixed stage animals in SDS-PAGE loading buffer for 5 minutes with subsequent centrifugation at high speed for 10 minutes. Lysates were loaded in SDS-gel PAGE and blotted with JMJD-1.2 (1:2500) and Actin (Millipore, MAB1501, 1:1000) antibodies. Polyclonal antibodies against JMJD-1.2 are described in ref.^15^.

### Germline immunofluorescence

Excised germlines were extracted from synchronized young adult (24 h post L4). For staining with antibodies against H3K9me2 (Abcam, ab1220), H3K27me2 (Abcam, ab24684) and phospho-histone H3 (Ser10) (Upstate 16–189) germlines were fixed for 5–10 minutes with 2% formaldehyde (Sigma Aldrich). Germlines were then freeze-cracked on dry ice, and then placed for 2 minutes in −20 °C methanol (Merk). For staining with antibodies against H3K23me2 (Active Motif, 39653), germlines were extracted, freeze-cracked, fixed for 2 minutes in −20 °C methanol (Merk), for 4 minutes in −20 °C acetone (Sigma-Aldrich) and then sequentially exposed to 95%, 70%, 50%, 30% ethanol solution (2 min each). For the JMJD-1.2 staining, germlines were fixed for 1 minute in 2% formaldehyde. After fixation, all the slides were washed for 15–30 minutes in PBST and blocked for 30 minutes to 1 hour in 1% BSA in PBST. After blocking, all of the aforementioned antibodies were diluted 1:200, except for JMJD-1.2 (1:1000), in blocking solution. Slides were placed in a humid chamber at 4 °C overnight and then washed 3 times 10 minutes in PBST. Secondary antibodies, donkey anti-mouse Alexa 488 (Life Technologies) and donkey anti-rabbit Alexa 594 (Life Technologies), were diluted 1:200 in blocking solution, incubated 2 hours at room temperature, and then washed 3 times 10 minutes in PBST. A drop of ProLong Gold Antifade Mountant with DAPI (Life Technologies) was added to slides, which were then sealed with a coverslip. Experiments were performed at least in duplicate. The specificity of the JMJD-1.2 antibody by IF was tested in ref.^15^ using mutant alleles.

### Imaging and quantification

Images (0.2 μm sections) were acquired at 40×, 60× and 100× magnification of optically bisected germlines^20^ using a Deltavision, deconvolved, and merged using softWorRx (Applied Precision). Exposure conditions were kept constant for each strain and condition. Germlines regions were determined as described^50^ and the fluorescence intensity for each region was measured using ImageJ (version 2.0.0) and normalized to the number of nuclei in the given region. 3–8 germlines were quantified for each strain and condition. Student’s *t* test statistical analyses were performed using GraphPad Prism. Images were further processed using ImageJ and Adobe Photoshop CS6. In Fig. 2, 78–302 mitotic cells, 59–125 transition zone cells and 60–323 pachytene cells were quantified. Quantitative analysis of RAD-51 foci presented in Fig. S5 was performed as described^51^.

### HU and irradiation exposures

Long HU treatment: synchronized young adult animals (24 h post L4) were placed for 16–19 h on freshly-prepared plates in which HU (Sigma Aldrich) was dissolved to a final concentration of 25 mM. NGM plates were seeded with a thin layer of OP50 the day before the use.

Short HU treatment: synchronized non-gravid young adult were placed for 5–6 h on freshly-prepared plates as described above.

For irradiation exposure, synchronized young adult animals were placed on seeded NGM plates and exposed to 80 Gy IR (Faxitron X-Ray LLC) or on unseeded NGM plates and exposed to 300 J/m^2^ UV-C (Dr. Groebel) and then put immediately onto plates containing OP50. *jmjd-5*(*tm3735*)^51^ and *xpa-1*(*mn157*)^52^ mutant alleles are used as positive controls for IR and UV tests, respectively. The experiments were done at least in triplicate.

### Embryonic lethality, cell cycle checkpoint, and apoptosis assays

Embryonic lethality was determined by exposing synchronized young adult animals to 25 mM HU for the indicated times and then putting 5–7 animals of each genotype on 3–4 plates for 5–6 h. For irradiation, synchronized young adult animals were treated and then rescued for 18 h, at which time 5 animals of each genotype were placed on 3 plates for 6 h. The number of dead embryos and living animals was counted 24 h later. Cell cycle arrest analysis in the mitotic region of germlines (N ≥ 15) was performed as described^20^. Apoptosis analysis in the meiotic germline (N ≥ 15) using CED-1::GFP was performed as described^29^. The experiments were done at least in triplicate, using synchronized young adult animals. Student’s *t* test statistical analyses were performed using GraphPad Prism. Cy3-dUTP (GE Healthcare) experiments were performed injecting the fluorescent nucleotide in synchronized young adult animals as described^19^ and at least six injected animals were analyzed for genotype and conditions.

### Phenotypic analyses

Brood size, embryonic lethality, and male production were determined by picking L4 animals of indicated genotypes to individual plates and passing daily. Approximately 24 h after, hatched and un-hatched progeny were counted. The number of males produced was counted after animals reached adulthood. Experiments were performed at 20 °C.

### Scoring of F2 generations after HU exposure

Young adult of N2 and *jmjd-1*.*2* mutants were treated with HU (25 mM, 16 h) and surviving F1 progeny were singled (n ≥ 30/experiment). The F2 generation was analyzed for gross physical defects, using a dissecting light microscope (Zeiss). The percentage of F2 abnormalities was determined based on the number of plates that had at least one F2 abnormal animal. The experiment was performed 2 times with the *jmjd-1*.*2*(*zr1010*) and 4 times with *jmjd-1*.*2*(*tm3713*), with similar results. Data presented in Fig. 5a are from two biological replicates. Chi-square test was used to evaluate statistical significance.

### Detection of mutations after HU exposure

The strain NL3400 carries multiple copies of the transgene pkIs1604 [HSP-16.2::ATG(A)17GFP/LacZ + pRF4(rol-6(su1006)], a stably integrated *lacZ* reporter construct under an heat shock promoter. The *lacZ* reporter is placed out of frame by an A17 mononucleotide DNA repeat inserted between the ATG and the *lacZ* open reading frame^32^. The DNA repeat enhances the chance of frame shift mutations to occur and reporter constructs that acquire mutations bringing the *lacZ* back in frame will result in blue patches in the animal body, after a X-gal staining. *jmjd-1*.*2*(*tm3713*) mutant animals carrying the pkIs1604 transgene were generated by standard crossing. Synchronized young adults of NL3400 (used as control) and *jmjd-1*.*2* carrying the pkIs1604 transgene were exposed to HU for 16 h, moved to normal plates and allowed to lay eggs (F1) for 6 h. At young adulthood, F1 animals were heat shocked at 34 °C (3 times for 40 minutes), briefly recovered, and stained for the presence of B-galactosidase with X-gal (5-bromo-4-chloro-3-indolyl-β-D-galactopyranoside). Two biological independent experiments were performed and Chi-square test was used to evaluate statistical significance.

## Electronic supplementary material

Supplementary information
