# Supplementary material for: JMJD-1.2 controls multiple histone post-translational modifications in germ cells and protects the genome from replication stress
Source: Sci Rep. 2018 Feb 28;8:3765. doi: 10.1038/s41598-018-21914-9 (PMC5830613; doi:10.1038/s41598-018-21914-9)
Supplement: Supplementary file 1 — Supplementary information [file 41598_2018_21914_MOESM1_ESM.pdf]

Supplementary information

JMJD-1.2 controls multiple histone post-translational modifications in germ cells and protects the genome from replication stress

Toshia R. Myers, Pier Giorgio Amendola, Yvonne C. Lussi and Anna Elisabetta Salcini

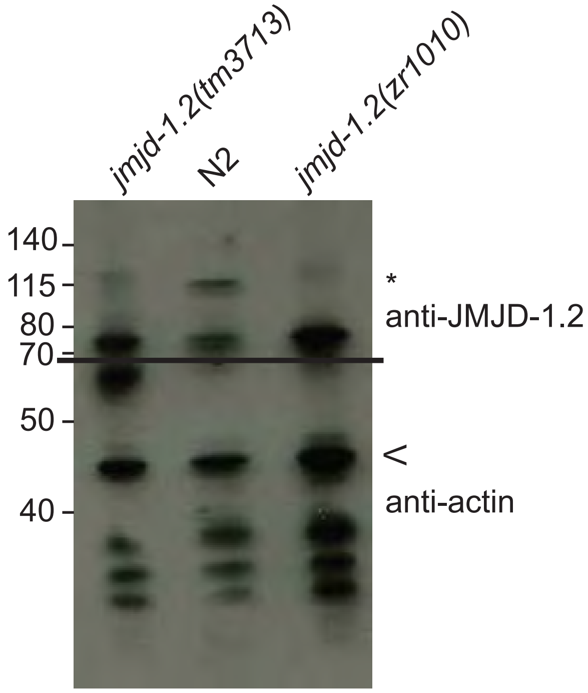
jmjd-1.2(tm3713)
N2
jmjd-1.2(zr1010)
140
115
80
70
50
40
*
anti-JMJD-1.2
<
anti-actin

Figure S1. JMJD-1.2 protein level in wild-type and *jmjd-1.2* mutant animals

Original image of the western blot presented in Fig. 1. The membrane was cut in two parts, as indicated, just below the 70 KDa band and blotted with anti-JMJD-1.2 (upper part) and actin antibodies (lower part). Markers are from Thermo Scientific (26616) and the gel, run with a MES buffer, is a NuPAGE 4-12% gradient gel from Invitrogen. Asterisk and arrowhead indicate JMJD-1.2 and actin bands, respectively.

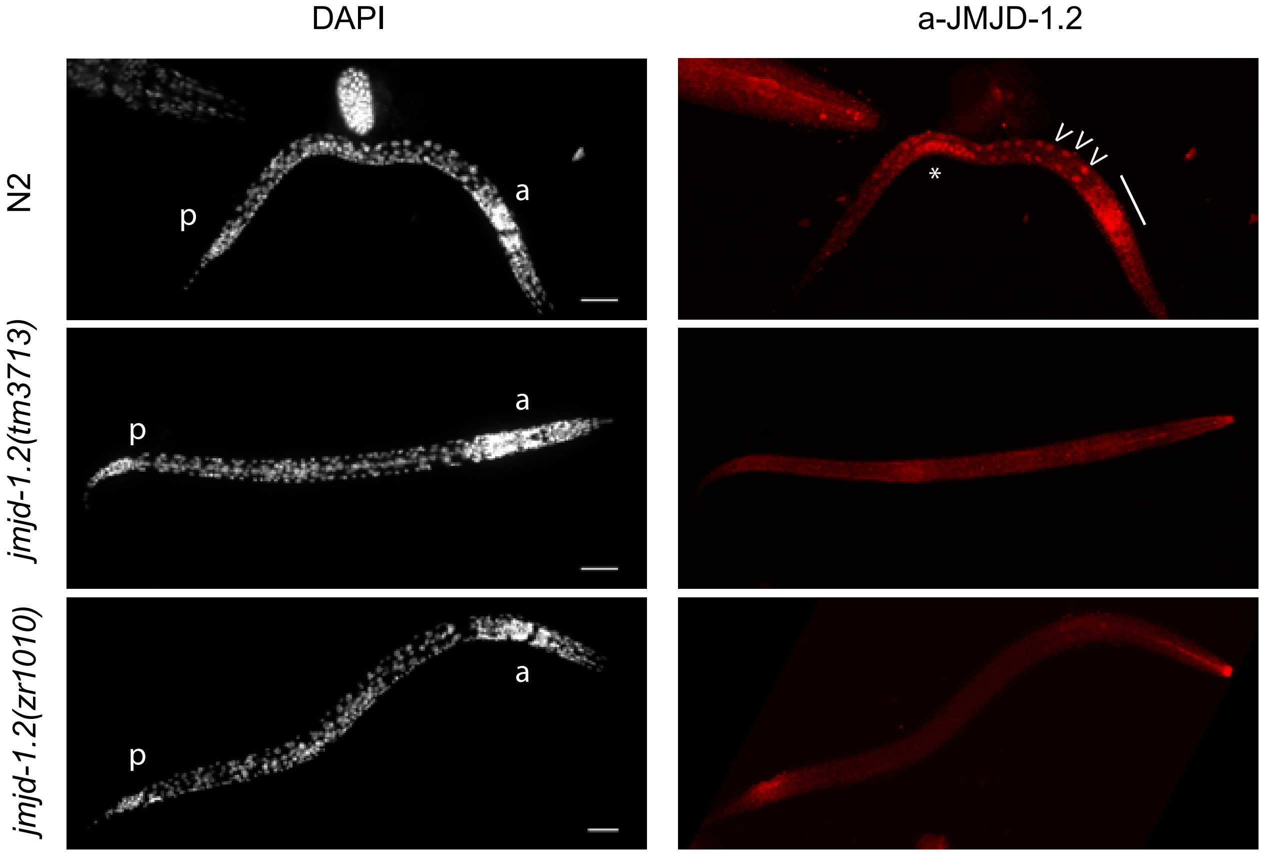

DAPI
a-JMJD-1.2
N2
p
a
*
jmjd-1.2(tm3713)
p
a
jmjd-1.2(zr1010)
a
p

Figure S2. JMJD-1.2 expression pattern in wild-type and *jmjd-1.2* mutant animals

Young animals (L2) of the indicated genotypes were stained with anti JMJD-1.2 antibody. Asterisk indicate the germline region and the arrowheads the intestine cells. The line points to the nerve ring region, enriched in neuronal cells. a, anterior, p, posterior of the animals. Animals are presented in a lateral view (ventral is down), with the exception of the middle panel, which is a ventral view. Scale bars, 25μm.

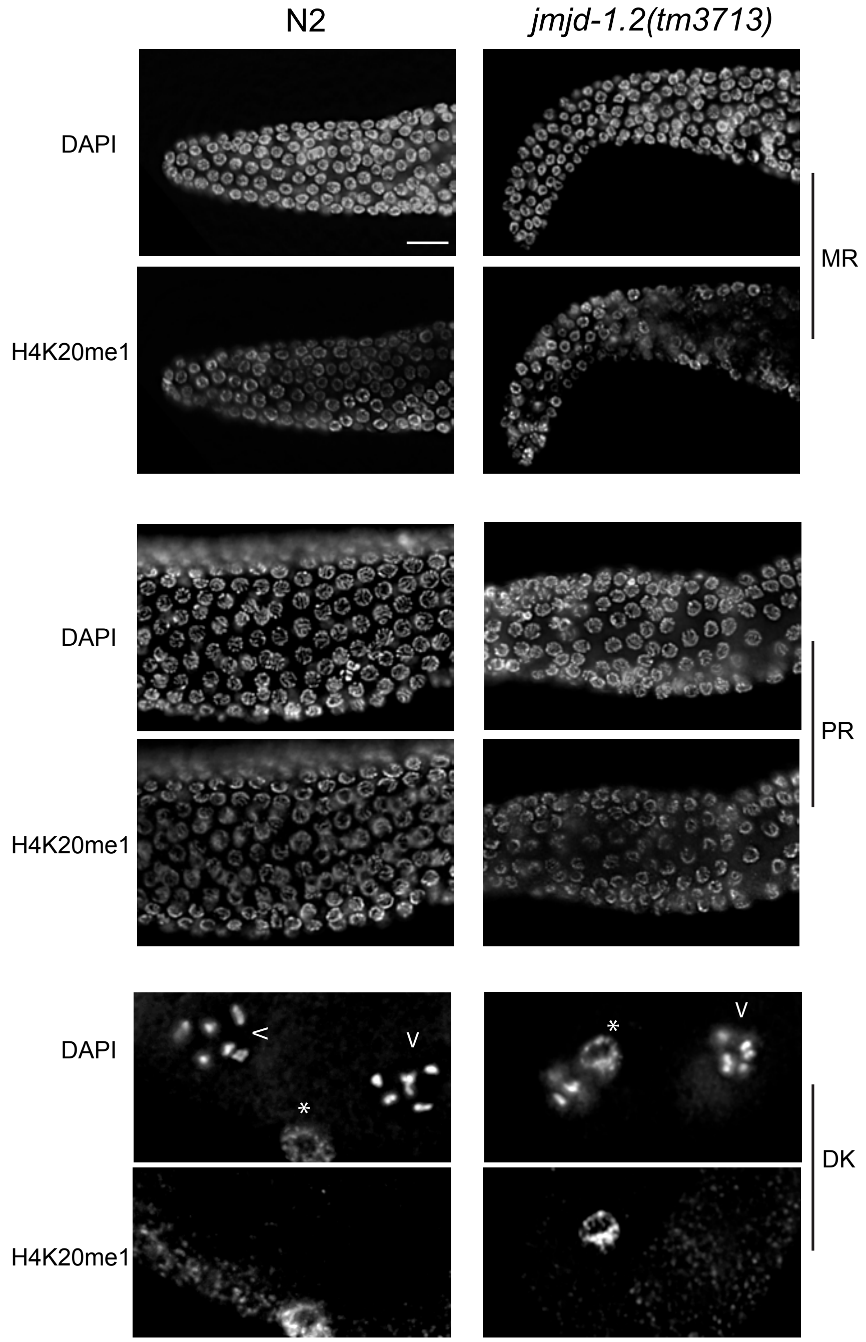

N2
jmjd-1.2(tm3713)
DAPI
H4K20me1
MR
DAPI
H4K20me1
PR
DAPI
H4K20me1
DK
<
V
*
*
V

Figure S3. H4K20me1 level in *jmd-1.2* mutant animals

Representative images of extracted germlines of N2 (left) and *jmjd-1.2(tm3713)* (right) stained with DAPI (top) and anti-H4K20me1(bottom). MR, mitotic region; PR, pachytene region; DK, oocytes in diakinesis (indicated by an arrowhead). Asterisks indicate somatic cells. Scale bar, 10μm

a

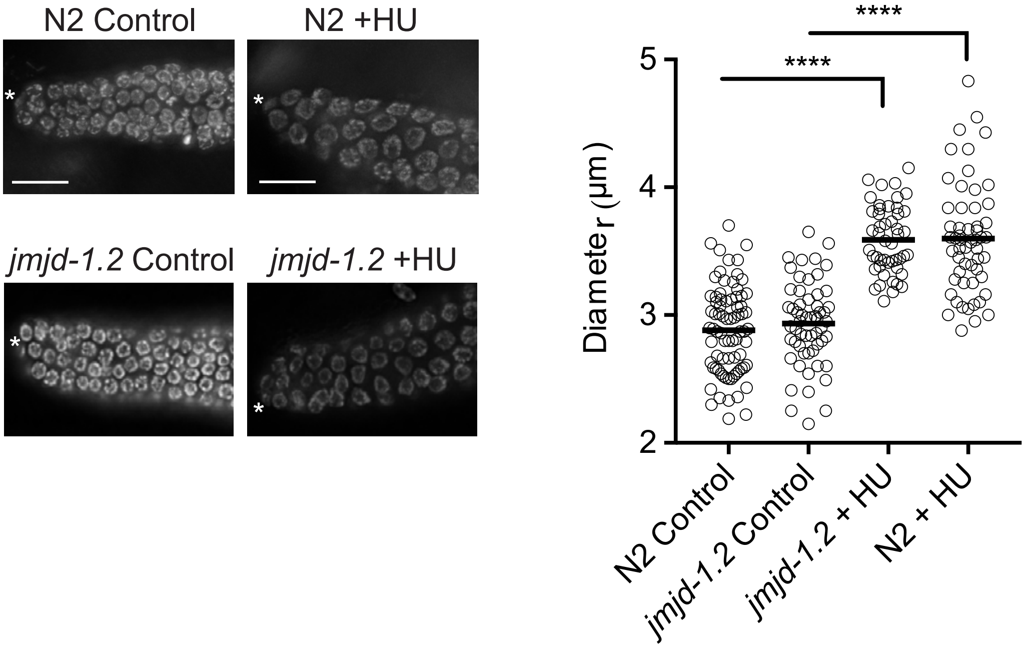

N2 Control
N2 +HU
jmjd-1.2 Control
jmjd-1.2 +HU
Diameter (µm)
5
4
3
2
****
****
N2 Control
jmjd-1.2 Control
jmjd-1.2 + HU
N2 + HU

b

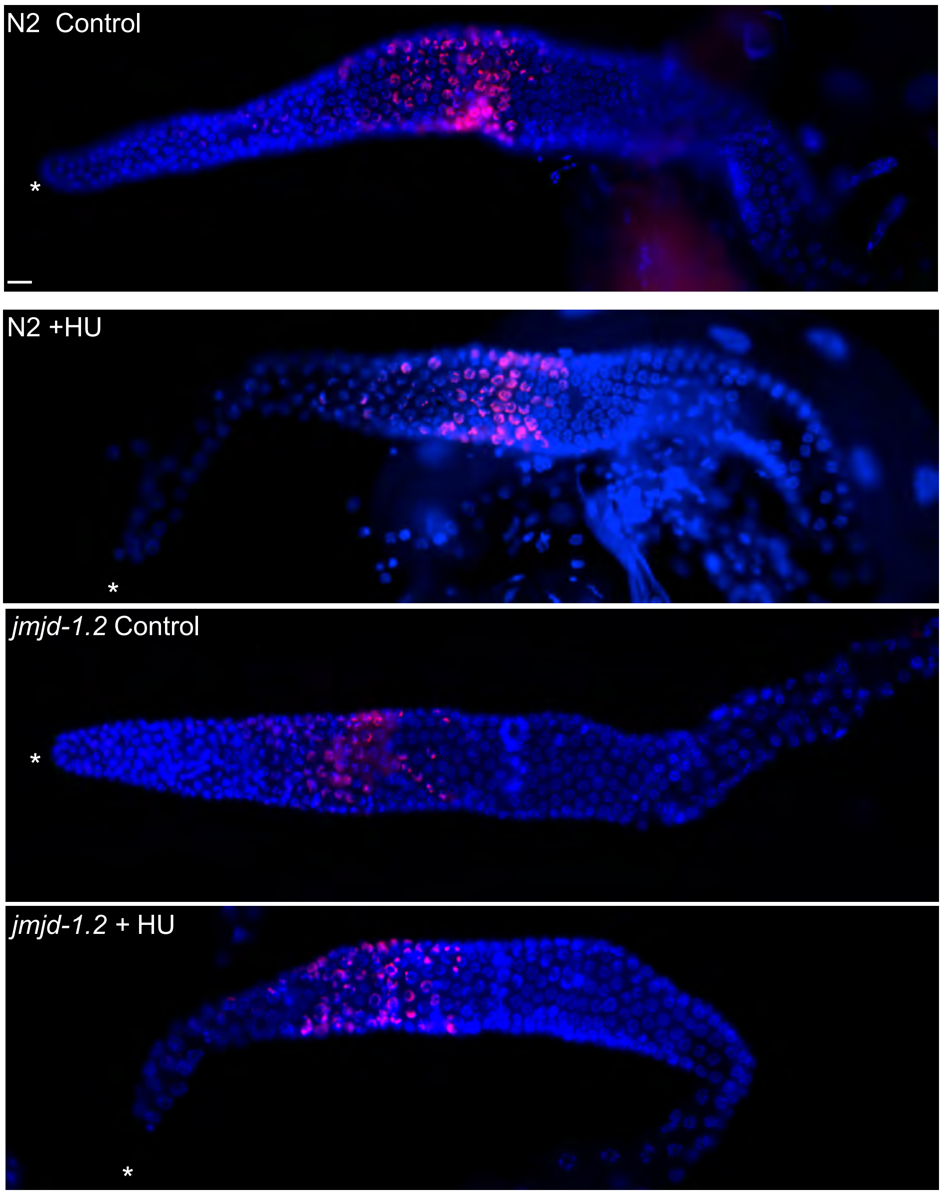

N2 Control
N2 +HU
jmjd-1.2 Control
jmjd-1.2 + HU

Figure S4. Mitotic cell arrest in *jmjd-1.2* mutant animals and germ cell progression after HU exposure

(**a**) Representative images of the mitotic region of extracted adult germlines from N2 and *jmjd-1.2(tm3713)* animals treated with HU (25mM, 16 hours) (+HU) or without treatment (Control) and stained with DAPI. Quantification of the mitotic cells diameter is given in the graphic on the right. Each circle represents a single nucleus and at least five gonads for genotype were analyzed. ****$p \leq 0.0001$, with unpaired *t*-test. (**b**) Representative images of extracted adult germlines from N2 and *jmjd-1.2(tm3713)* animals treated with HU or without treatment. Animals were injected with Cy3-dUTP, exposed to 25mM HU for 20 hours, and the gonads dissected, fixed and stained with DAPI. After 20 hours, Cy3-dUTP-positive cells (in red) are in pachytene. Asterisks indicate the distal part of the germlines. Scale bars, 10μm.

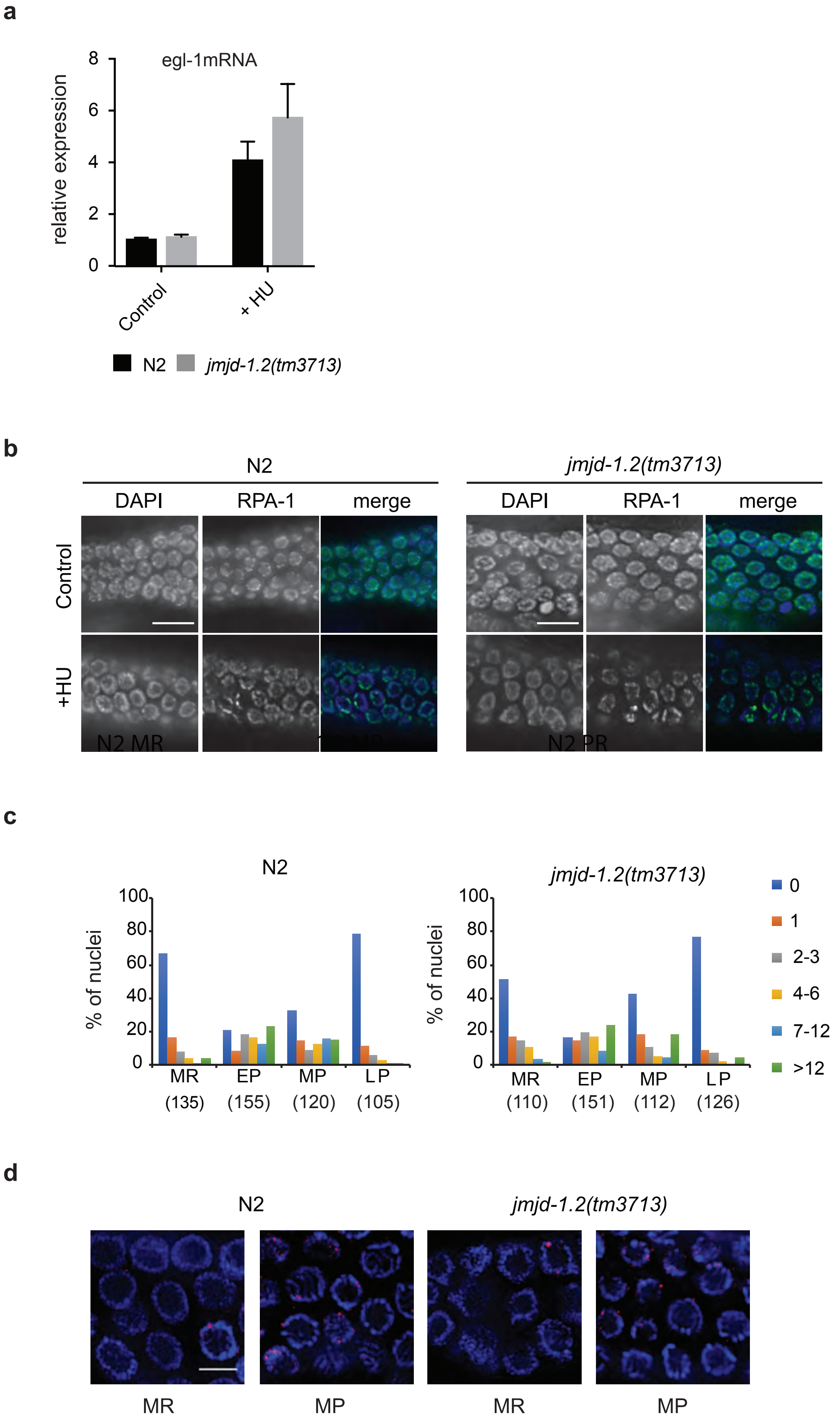
a
egl-1mRNA
relative expression
0
2
4
6
8
Control
+ HU
N2
jmjd-1.2(tm3713)
b
N2
jmjd-1.2(tm3713)
DAPI
RPA-1
merge
DAPI
RPA-1
merge
Control
+HU
N2 MR
N2 PR
c
N2
jmjd-1.2(tm3713)
% of nuclei
0
20
40
60
80
100
MR
(135)
EP
(155)
MP
(120)
LP
(105)
MR
(110)
EP
(151)
MP
(112)
LP
(126)
0
1
2-3
4-6
7-12
>12
d
N2
jmjd-1.2(tm3713)
MR
MP
MR
MP

Figure S5. egl-1 level, RPA and RAD-51 recruitment in *jmjd-1.2* mutant animals

(**a**) Relative mRNA expression levels of the pre-apoptotic gene *egl-1* in N2 and *jmjd-1.2(tm3713)* mutant animals treated with 25mM HU, 16h (+HU) or without treatment (control). *rpl-26* is used as internal control. Bar indicates SD from technical triplicates. (**b**) Representative images of mitotic regions of germlines excised from N2 and *jmjd-1.2(tm3713)* animals carrying an integrated RPA-1::YFP transgene, treated with HU (25mM, 16 hours) (+HU) or without treatment (Control). DAPI staining (blue) in the left panels and anti-GFP staining (green) in the middle panels. Scale bar, 10μm. (**c**) Histogram showing the quantification of RAD-51 foci in different regions of N2 and *jmjd-1.2(tm3713)* germlines after HU treatment (25mM, 16h). MR, mitotic region; EP, early pachytene; MP, medium pachytene; LP, late pachytene. Numbers in parenthesis indicate the number of nuclei analyzed. At least five gonads were analyzed per genotype. No significant differences were observed with two-tailed paired *t*-test ($p > 0.1$). (**d**) Representative images of RAD-51 staining of N2 and *jmjd-1.2* mitotic (MR) and medium pachitene (MP) regions after HU treatment (25mM, 16h). Scale bar, 5 μm.
